# Phenotyping EMT and MET cellular states in lung cancer patient liquid biopsies at a personalized level using mass cytometry

**DOI:** 10.1038/s41598-023-46458-5

**Published:** 2023-12-08

**Authors:** Loukia G. Karacosta, Danny Pancirer, Jordan S. Preiss, Jalen A. Benson, Winston Trope, Joseph B. Shrager, Arthur Wai Sung, Joel W. Neal, Sean C. Bendall, Heather Wakelee, Sylvia K. Plevritis

**Affiliations:** 1 Department of Biomedical Data Science, Stanford University, Stanford, CA 94305 USA; 2 Department of Cancer Systems Imaging, The University of Texas MD Anderson Cancer Center, Houston, TX 77054 USA; 3 Stanford Cancer Institute - Clinical Trials Office, Stanford University, Stanford, CA 94305 USA; 4 Department of Cardiothoracic Surgery, Stanford University, Stanford, CA 94305 USA; 5 Palo Alto VA Health Care System, Palo Alto, USA; 6 Division of Pulmonary, Allergy & Critical Care Medicine, Department of Medicine, Stanford University, Stanford, CA 94305 USA; 7 Division of Oncology, Department of Medicine, Stanford University, Stanford, CA 94305 USA; 8 Department of Pathology, Stanford University, Stanford, CA 94305 USA; 9 Department of Radiology, Stanford University, Stanford, CA 94305 USA

**Keywords:** Proteomic analysis, Lung cancer, Tumour biomarkers, Tumour heterogeneity, Functional clustering, Machine learning, Protein analysis, Cancer, Computational biology and bioinformatics, Systems biology, Biomarkers

## Abstract

Malignant pleural effusions (MPEs) can be utilized as liquid biopsy for phenotyping malignant cells and for precision immunotherapy, yet MPEs are inadequately studied at the single-cell proteomic level. Here we leverage mass cytometry to interrogate immune and epithelial cellular profiles of primary tumors and pleural effusions (PEs) from early and late-stage non-small cell lung cancer (NSCLC) patients, with the goal of assessing epithelial-mesenchymal transition (EMT) and mesenchymal-epithelial transition (MET) states in patient specimens. By using the EMT–MET reference map PHENOSTAMP, we observe a variety of EMT states in cytokeratin positive (CK+) cells, and report for the first time MET-enriched CK+ cells in MPEs. We show that these states may be relevant to disease stage and therapy response. Furthermore, we found that the fraction of CD33+ myeloid cells in PEs was positively correlated to the fraction of CK+ cells. Longitudinal analysis of MPEs drawn 2 months apart from a patient undergoing therapy, revealed that CK+ cells acquired heterogeneous EMT features during treatment. We present this work as a feasibility study that justifies deeper characterization of EMT and MET states in malignant cells found in PEs as a promising clinical platform to better evaluate disease progression and treatment response at a personalized level.

## Introduction

The epithelial-mesenchymal transition (EMT) and the reverse process of mesenchymal-epithelial transition (MET) have been considered key processes of tumor plasticity in the metastatic cascade^1^ and drug resistance^2,3^, with MET regarded as a critical step for distant metastasis^4,5^. Despite numerous in vitro investigations supporting the relevance of EMT and MET plasticity in cancer, controversies of its relevance to clinical disease remain due to scarcity of fully mesenchymal cells detected in clinical specimens, and the fact that epithelial cells are also capable of displaying metastatic capabilities^6,7^. The transient and dynamic natures of EMT and MET, along with the difficulties in acquiring patient serial biopsies, especially of metastatic lesions, make it challenging to address EMT-related controversies, particularly in the case of MET.

Single-cell approaches have enabled a richer characterization of EMT-enriched phenotypic states of malignant cells in clinical specimens, often highlighting the prevalence of partial EMT (pEMT) states whereby malignant cells co-express epithelial and mesenchymal features^8,9^. MET however, has yet to be well documented in clinical specimens. In fact, clinical occurrence of MET has been questioned by observational studies that show malignant cells at metastatic sites are phenotypically similar, even in terms of their EMT state, to their matched primary tumor^10^. To provide a profile of EMT and MET states in clinical samples, we recently defined a spectrum of EMT states exhibited by non-small cell lung cancer (NSCLC) cell lines in a time-course study under TGFβ treatment and withdrawal. With these states, we constructed a 2D map, PHENOtypic STAte MaP (PHENOSTAMP), through which EMT status of clinical specimens can be assessed using single-cell proteomic measurements^11^. Our study identified a distinct MET state, which we only observed when the cells were returning to the epithelial state during TGFβ withdrawal. Consistent with other studies, we found that malignant cells in primary resected lung adenocarcinomas primarily exhibited epithelial and pEMT states but did not exhibit MET. This corroborates the hypothesis that MET takes place in advanced metastatic and/or treatment refractory lung cancer.

Given the challenges in obtaining metastatic tissue from advanced stage patients, we sought to analyze malignant pleural effusions (MPEs) from late-stage NSCLC patients. MPEs occur in approximately 30% of lung cancer cases and are associated with poor prognosis^12,13^. The standard approach for MPE diagnosis is cytological analysis, although detection remains limited due to scarcity of malignant cells and difficulty in distinguishing them from other cell types. Despite these limitations, studies have proposed harnessing MPEs as a liquid biopsy for phenotyping malignant cells^14–16^ and guiding precision immunotherapy^17^. Malignant cells found in pleural effusions (PEs) have been described as phenotypically similar to circulating tumor cells (CTCs), however instead of travelling in circulation through the vascular system, they float within the pleural cavity^15^ and in certain cases are able to metastasize across the pleural cavity, a phenomenon termed as transcoleomic spread^18^. Given the established roles of EMT and MET in lung cancer metastasis, therapy resistance and poor outcome^2,3,19^, and because MPEs are often drained from patients as palliative care, our study of lung cancer MPEs offers a unique opportunity to delineate the dynamic, EMT and MET- related changes in cells during metastasis and therapy at a personalized level^20,21^.

MPEs are inadequately studied at the single-cell level. Here, we utilize mass cytometry to phenotypically interrogate ~2000 cytokeratin positive (CK+) cells and ~ 90,000 immune cells in PEs from 8 late-stage NSCLC patients and contrast the results with 5 primary tumors from early-stage patients. By projecting CK+ cells on PHENOSTAMP^11^, we observe a variety of EMT phenotypic states, and also report the detection of CK+ cells in an MET phenotypic state. We also simultaneously characterize immune cell types of PEs and found that the presence of CD33+ myeloid cells co-occur with CK+ cells.

## Results

### Mass cytometry study design of primary lung tumors and PEs enables single-cell analysis of CK+ and immune cell subpopulations

**Figure 1:**
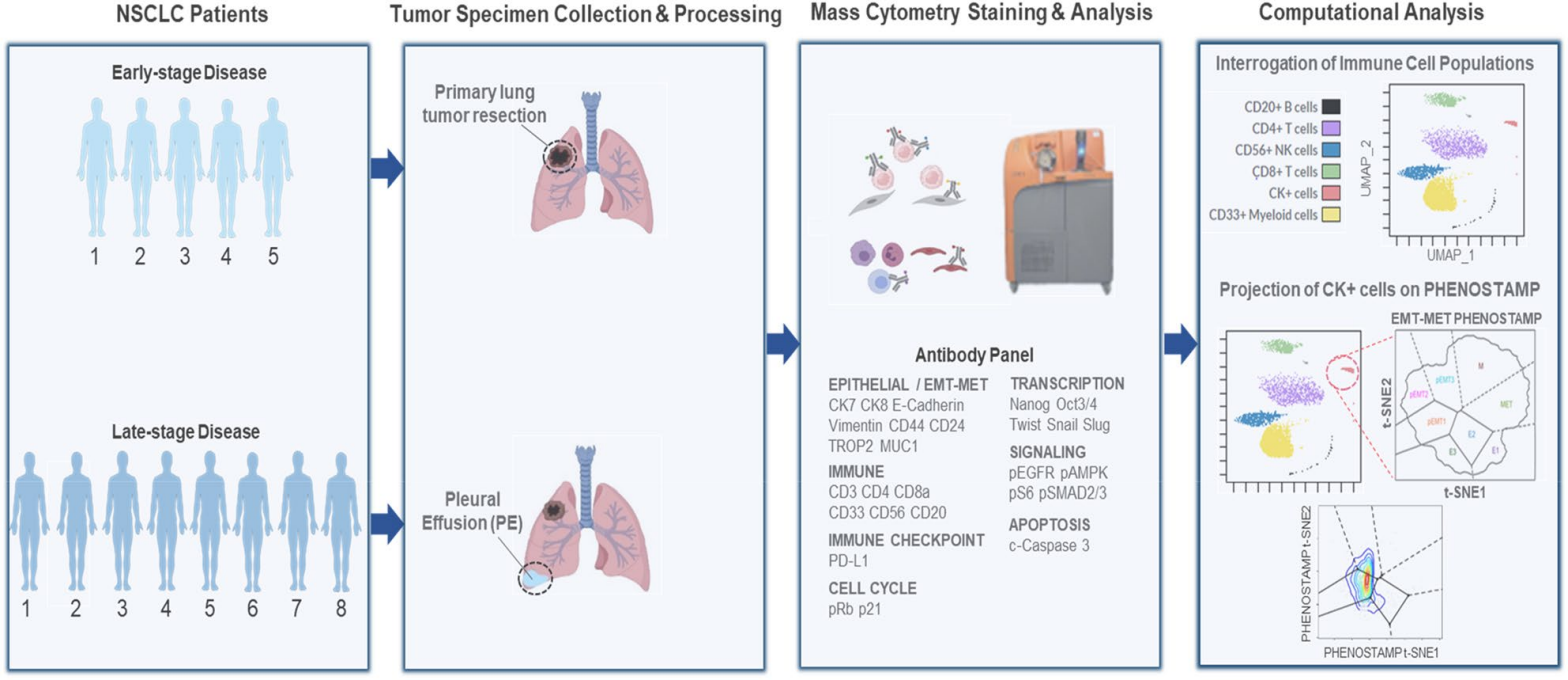
Schematic of study design and workflow. First and second panel: Freshly resected primary tumors and PE clinical specimens were obtained from five and eight, respectively, NSCLC patients. Third panel: Mass cytometry analysis of clinical specimens. Primary solid tumors were dissociated into single cell suspensions as previously described^11^, prior to processing for mass cytometry staining and analysis. Antibodies specific to stromal (FAP+), endothelial cells (CD31+), cytokeratin positive (CK+) cells and immune subpopulations were used for manual gating and downstream analysis. Shown here is the common panel of antibodies that was used to phenotype CK+ and immune cells in both primary tumors and PEs. In some cases, additional antibodies were used in separate runs for further specimen characterization. Fourth panel: UMAP was utilized to visualize the cellular profiles of all primary tumor and PE clinical specimens. CK+ cells were further assessed for EMT/MET status by projecting on the PHENOtypic STAte MaP (PHENOSTAMP) developed previously by our group^11^. Additional details can be found in the Materials and Methods section, in Supplementary Tables 1, 2 and Supplementary Fig. 1.

Clinical research specimens were obtained following resection of primary tumors and removal of PEs under Institutional Review Board (IRB) approval. To identify and compare CK+ and immune cell types in (i) primary tumors from early-stage NSCLC patients, and (ii) PE clinical specimens from late-stage NSCLC patients, we developed a mass cytometry panel with antibodies that were selected to identify epithelial cells along a spectrum EMT phenotypic states and five main immune populations, specifically: CD4+ T cells, CD8+ T cells, CD33+ Myeloid cells, CD56+ NK cells and CD20+ B cells. Cytokeratins 7 and 8 were used to detect epithelial cells, and markers such as E-Cadherin, Vimentin, CD44, CD24 and MUC1 were included to delineate EMT status as previously described by utilizing PHENOSTAMP^11^. UMAP analysis^22^ was used to visualize immune cell types of each clinical specimen. Figure 1 illustrates the study design, including the common antibodies that were used in all mass cytometry runs across all specimens. Additional antibodies that were used for specific analyses are described throughout the text and in the supplementary documentation (Fig. 1, Supplementary Table 1, Supplementary Fig. 1).

### CK+ cells in PEs, but not primary lung tumors, demonstrate enrichment of the MET state

**Figure 2:**
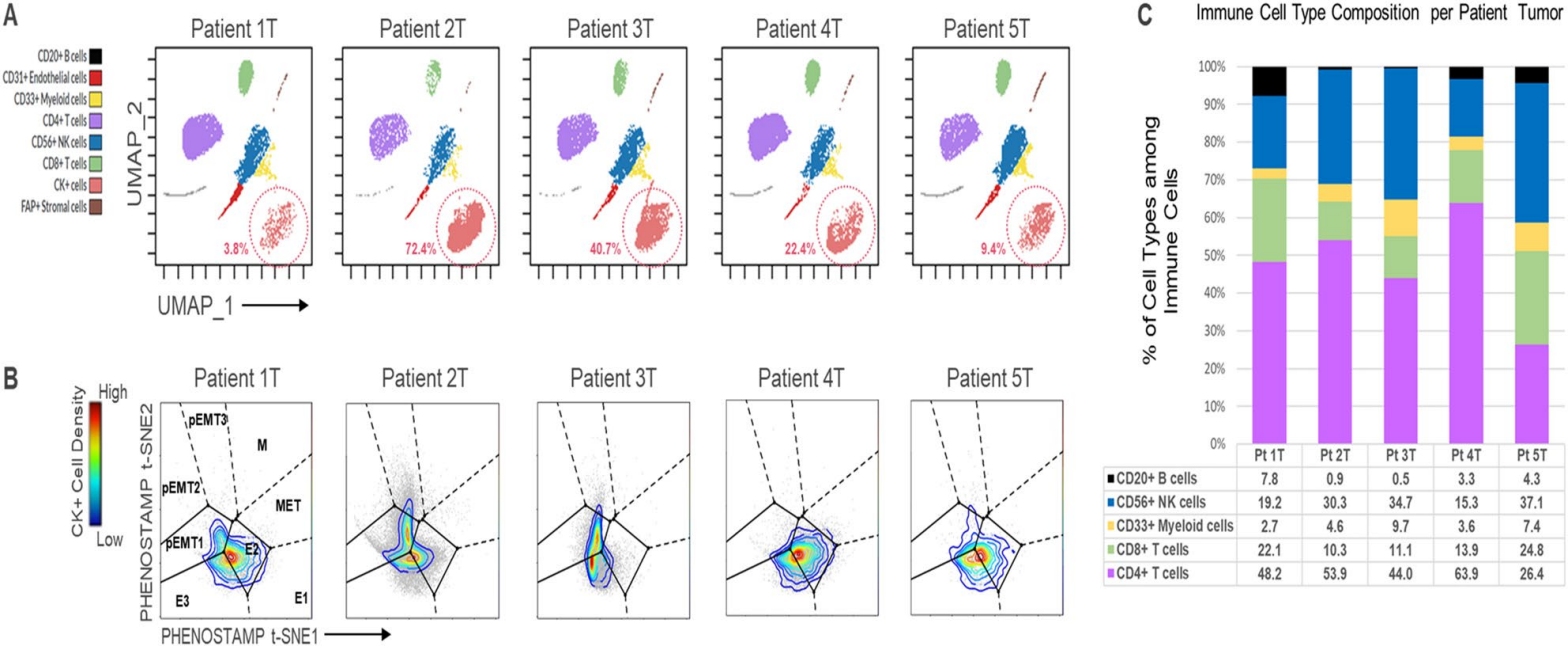
EMT/MET phenotypic analysis of CK+ cells and immune cells derived from primary tumor (T) of NSCLC patients and analyzed by mass cytometry. (**A**) UMAP of the mass cytometry data generated on each of the 5 resected primary tumors, with n = 10,000 randomly sampled cells per tumor. Each dot represents a single cell colored by its cell type as derived from manual gating and UMAP analysis of the mass cytometry data. Dotted circles indicate percentage of CK+ cells per tumor (per 10,000 tumor cells in our UMAP analysis). (**B**) Projections of manually gated CK+ cells from each patient tumor specimen onto PHENOSTAMP to assess EMT and MET status^11^. Number of projected cells per patient tumor 1T–5T: 4262, 41760, 17991, 8803, 1614, respectively. Three of the five samples, namely 1T, 4T and 5T are regarded as Epithelial (E)-enriched tumors; whereas, two of the 5 samples, namely 2T and 3T, are regarded as pEMT-enriched tumors. (**C**) Immune cell type composition per tumor specimen. Each immune cell type was calculated as percentage of total number of immune cells per patient (Pt) tumor, as defined by the UMAP analysis. CD20+ B cell and CD8+ T cell proportions were lower in the pEMT-enriched tumors (2T, 3T), compared to the Epithelial (E)-enriched tumors (1T, 4T, 5T), whereas CD33+ myeloid cells and CD56+ NK cells were higher (*p* < 0.001, chi-square test). CD4+ T cells were not significantly different among the E- versus pEMT-enriched tumors. See Materials and Methods, Supplementary Table 1 and Supplementary Figs. 1 and 2 for additional information).

*Primary Tumors:* We sought to interrogate EMT states and immune cell types of five primary, treatment-naive NSCLC tumors (Supplementary Table 2) through single-cell analysis via mass cytometry. This analysis is similar to our prior work^11^, but, unlike our prior work, this analysis also includes characterization of the non-malignant cells in the tumor microenvironment. From UMAP analysis, we highlight the CK+ cells as distinct from the immune, endothelial and stromal subpopulations (Fig. 2A, Supplementary Fig. 2). Manually gated CK+ cells were projected onto PHENOSTAMP to assess their EMT/MET phenotype (Fig. 2B) as previously described^11^. In all primary specimens, the CK+ cells occupied E and pEMT states (primarily pEMT1) (Fig. 2B). Specifically, Specimens 1T, 4T and 5T comprised of mostly cells in E states and Specimens 2T and 3T comprised cells that spanned both E and pEMT states. We did not detect significant numbers of CK+ cells in the M and MET states—a finding which is consistent with our previous study of similar tumor specimens^11^. However, unlike our prior work, here we also investigated the immune cells alongside the epithelial cells. Overall, when we compared the pEMT-enriched (2T, 3T) versus the E-enriched tumors (1T, 4T, 5T), we found that CD20+ B cell and CD8+ T cell proportions were lower in the pEMT-enriched tumors, whereas CD33+ myeloid cells and CD56+ NK cells were higher (*p* < 0.001, chi-square test) (Fig. 2C). The fraction of CD4+ T cells were not significantly different among the pEMT- versus E-enriched tumors.

**Figure 3:**
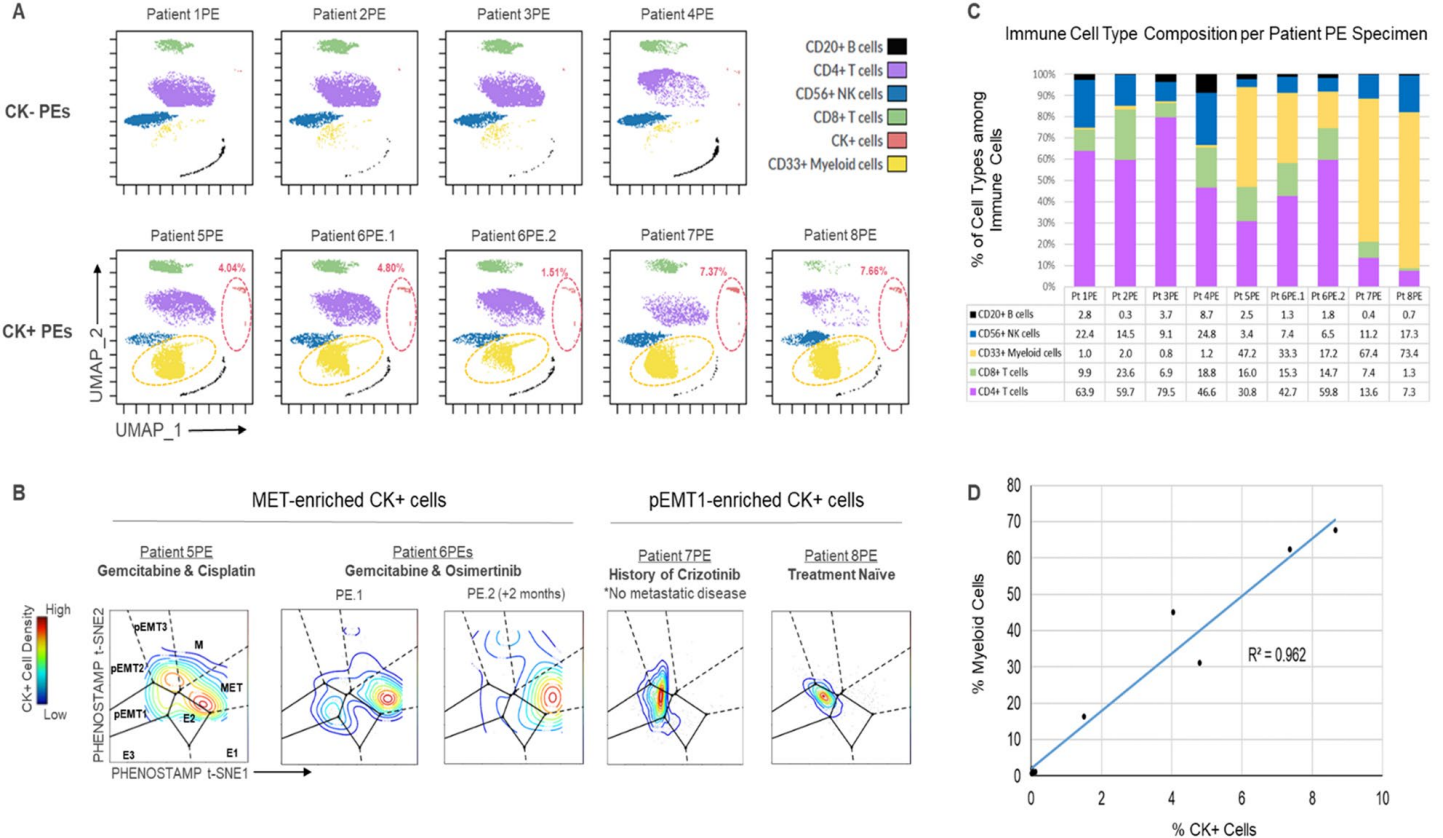
Cellular phenotyping of PEs identifies CK+ cells in the MET state. (**A**) UMAP of each PE specimen (n = 10,000 randomly sampled cells per specimen). Top row shows PEs where we did not detect a significant number of CK+ cells, (aka, the “CK−“ PE group). Bottom row shows PEs where we detected a significant number of CK+ cells (“CK+ ” PE group). Each dot represents a single cell colored by its cell type as defined by manual gating and UMAP analysis of the mass cytometry data. Dotted red circles indicate percentage of CK+ cells per PE specimen. Dotted yellow circles highlight the observed increased presence of CD33+ myeloid cells in the CK+ PE group of specimens. (**B**) Projections of manually gated CK+ cells from each CK+ PE specimen onto PHENOSTAMP. Note the high density of cells mapping onto the MET state in specimens from Patients No5 and 6. Number of projected CK+ cells per specimens 5PE-8PE: 58, 284, 39, 879, 751 respectively. (**C**) Immune cell type composition per PE specimen. Each immune cell type was calculated as percentage of total number of immune cells per PE. (**D**) Percentage of CK+ cells in PEs shows positive correlation with percentage of CD33+ myeloid cells in all specimens. See Materials and Methods and Supplementary Figs. 3–6 for additional information.

*PE Specimens:* We obtained and analyzed 9 PE specimens from 8 late-stage NSCLC patients. All patients but one had undergone treatment for advanced disease (Fig. 1, Table 1). For one patient, we were able to acquire 2 longitudinal PE specimens, drawn 2 months apart. Our mass cytometry analysis was in agreement with the cytological reports in 7 out of 9 specimens (Table 1). Among the 7 PE specimens which were reported positive for malignant cells with conventional cytology, 5 specimens presented CK+ cells with mass cytometry analysis and 2 did not. Among two PE specimens which were reported negative for malignant cells with conventional cytology, both reported negative with mass cytometry. The immune composition of the CK- PE patients was more similar with that in the primary tumor of patients with early-stage disease than that of patients with CK+ PEs (See Fig. 2C and part of Fig. 3C).

**Table 1:** Clinical data on PE specimens: For the 9 PE specimens from 8 late-stage NSCLC patients, data on Clinical Information (left panel) and PE Processing Results (right panel) are provided.

Clinical information							PE processing results		
Patient #	Cancer type	Stage	Mutation	Treatment at time of PE collection	Cytology report for presence of malignant cells per PE	PE volume (mL)	# of recovered viable cells	% Viability	Detection of CK+ cells with Mass cytometry
1	NSCLC, Adenocarcinoma	IV, T4NxM1b	MET e14 Skip & TP53	Carboplatin-Pemetrexed	Negative	50	25.4 × 10^6^	N/A	No
2	NSCLC, Adenocarcinoma	IV, T1aN3M1c	N/A	Pemetrexed-Pembrolizumab	Negative	60	2.7 × 10^6^	98.8	No
3	NSCLC, Adenocarcinoma & Colon Cancer	I, IV	EGFR, ALK+	Lorlatinib	Positive	60	31 × 10^6^	98.8	No
4	NSCLC, Adenocarcinoma	IV, T1N0M1	EGFR e19	Osimertinib	Positive	60	5.8 × 10^6^	95	No
5	NSCLC, Adenocarcinoma	IV, T4N3M1b	EGFR L858R	Gemcitabine-Cisplatin	Positive	60	4 × 10^6^	91.6	Yes
6	NSCLC, Adenocarcinoma	IV, T2aN2M1b	EGFR e21L851Q	Gemcitabine-Osimertinib	Positive	65	30 × 10^6^	87	Yes
> >	> >	> >	> >	Gemcitabine-Osimertinib	Positive	70	3 4 × 10^6^	95.9	Yes
7	NSCLC, Adenocarcinoma	IIIA, T4N0M0	ROS1 rearrangement	Crizotinib (2 months prior)	Positive	50	10 × 10^6^	96	Yes
8	NSCLC, Adenocarcinoma	IV, T1N3M1	N/A	Treatment Naïve	Positive	58	52.3 × 10^6^	94.7	Yes

*PE* Pleural Effusion, *CK+ *Cytokeratin positive.

### CK+ cells in PEs exhibit pEMT and MET phenotypic features

Based on our analysis on primary tumor samples in this study and our prior study^11^, and reports showing EMT prevalence in CTCs^23,24^, we hypothesized that CK+ cells in PEs also visit a spectrum of EMT states, and, in contrast with primary tumors, a significant proportion of these cells exhibit MET features. Our rationale is that in advanced stage disease, malignant cells will manifest EMT-like properties that will deem them more “fit” for metastasis and drug resistance, as has been previously suggested in CTCs and in-vitro experiments^24,25^. Figure 3A shows the cell types of each PE specimen following mass cytometry analysis, where the top panels are the CK- samples (Patients Nos 1PE–4PE) and the bottom panels are the CK+ samples (Patients Nos 5PE-8PE). The proportion of CK+ cells detected in specimens from Patients No 5PE-8PE, ranged from 1.5 (Patient No 6PE.2) to 7.7% (Patient No 7PE) (Supplementary Figs. 3 and 4). When we projected CK+ cells from Patients Nos 5PE-8PE onto PHENOSTAMP (Fig. 3B), we observed that PE CK+ cells predominately mapped onto 2 distinct states: (i) the partial, pEMT1 state (Patients No 7PE and 8PE), consistent with reports of CTCs circulating in a partial/hybrid EMT state^25,26^; and (2) the MET state (Patients No 5PE and 6PE), a finding yet to be reported from clinical specimens. When we assessed the treatment status of the patients with CK+ cells, we found that Patients No5 and No6 were undergoing Gemcitabine-Cisplatin and Gemcitabine-Osimertinib therapy respectively, whereas in the case of the pEMT-enriched specimens, Patient No8 was treatment naïve and Patient No7 had last had treatment (Crizotinib) 2 months prior to specimen acquisition. Furthermore, Patient No7 was the only patient who had no known metastatic disease at the time the PE was collected and was stage IIIA (vs all other patients who were stage IV). pEMT1 cells expressed high E-Cadherin and moderate Vimentin levels and were heterogeneous for MUC1. Conversely, all MET cells expressed high levels of both Vimentin and MUC1 but were negative for E-Cadherin (Supplementary Fig. 5). The expression levels of these canonical EMT markers E-Cadherin, Vimentin and MUC1 in the pEMT1 versus MET states were in agreement with our previous observations in NSCLC cell lines^11^ (Supplementary Fig. 5); moreover, MUC1 was the main marker that signified the Mesenchymal (M) to MET switch upon TGFβ withdrawal in NSCLC cell lines in our previous study^11^. All these observations indicate the existence of an MET state in clinical samples.

### CK+ cells co-occur with CD33 + myeloid cells in PEs

Immune cell type composition per PE patient specimen is shown in Fig. 3C. The most prominent immune cell type was CD4+ T cells in all the CK- PEs (Range of CD4+ T percentages: 46–79%), whereas among the CK+ PEs, this was the case only in Patient No6 PE. CD8+ T cells were observed in all PEs with percentages ranging from 1.3% (Patient No8 PE) to 22.8% (Patient No2 PE). Although CD20 + B cell percentages were low and variable across all PEs, B cells in CK+ PEs showed significantly higher expression of phospho-Rb (pRB) compared to B cells in CK- PEs (p < 0.001, Student’s *t* test) (Supplementary Fig. 6). The most striking difference between CK+ and CK- PEs was the presence of CD33 + myeloid cells: we observed that a positive correlation of 0.96 between the fraction of CK+ cells and the fraction CD33 + myeloid cells (Fig. 3A, C and D).

### Differential immune cellular composition between pEMT-enriched versus MET-enriched CK+ PEs

**Figure 4:**
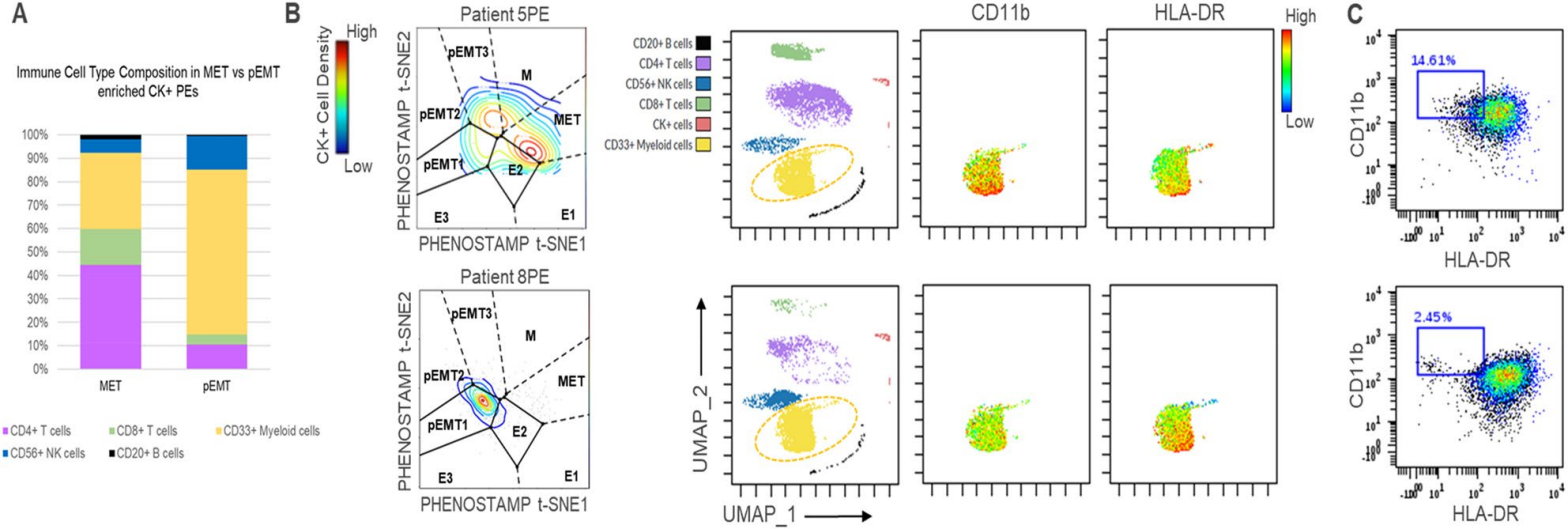
Comparison of immune cells in MET- versus pEMT-enriched CK+ PE specimens. (**A**) Averaged percentage of immune cell types in MET-enriched CK+ PEs (specimens 5PE, 6PE.1, 6PE.2) and pEMT-enriched CK+ PEs (specimens 7PE and 8PE). (**B**) For Patient No5 (MET-enriched, top panel) and No8 (pEMT-enriched, bottom panel): (Left panel) Projection of CK+ cells on PHENOSTAMP; (three right panels) UMAP of all defined cell populations, alongside expression profiles of CD11b and HLA-DR markers in the CD33+ population of myeloid cells. (**C**) CD11b/HLA-DR biaxial plots of CD33+ cells shows an increase of CD11b+ HLA-DR- myeloid cell percentage in specimen 5PE (top panel) versus 8PE (bottom panel), suggesting presence of myeloid-derived suppressor cells (MDSCs) in MET-enriched CK+ PEs.

Despite the small number of CK+ PEs we analyzed, we observed differences between the MET-enriched CK+ PEs (5PE, 6PE.1, 6PE.2) compared to the pEMT-enriched CK+ PEs (7PE and 8PE) (Figs. 3C and 4A). The MET-enriched CK+ PEs had lower percentages of CD56+ NK cells (3.4–6.3% for MET versus 11.2–17.3% for pEMT) and CD33 + myeloid cells (16.6–47% for MET versus 67.3–73.4% for pEMT). Conversely, they had higher percentages of CD4 T cells (30.7–57.6% for MET versus 7.3–13.6% for pEMT), CD8 T cells (14.2–16% for MET versus 1.3–7.4% for pEMT) and CD20 B cells (1.3–2.5% for MET versus 0.4–0.7% for pEMT). All observed differences in the immune populations were statistically significant (*p* < 0.001, chi-square test). Figure 4A represents the average percentage of each immune cell type in MET versus pEMT CK+ PEs.

### Evidence of myeloid-derived suppressor cells (MDSCs) in an MET-enriched CK+ PE

Given the immunosuppressive role of MDSCs^27^ and the correlation we observed between CK+ cells and CD33+ myeloid cells in PEs (Fig. 3D), we examined whether we could identify MDSCs (characterized as CD33+ CD11b+ HLA-DR-) in the CK+ PEs we analyzed. Interestingly, we observed increased number of cells with an MDSC phenotype in one of the MET-enriched CK+ PEs (5PE) when compared to one of the pEMT-enriched CK+ PE (8PE) (Fig. 4B, C), even though percentage of CD33+ myeloid cells was higher in patient No8 (see Fig. 3C).

### Longitudinal analysis suggests EMT/MET-related phenotypic changes during treatment

**Figure 5:**
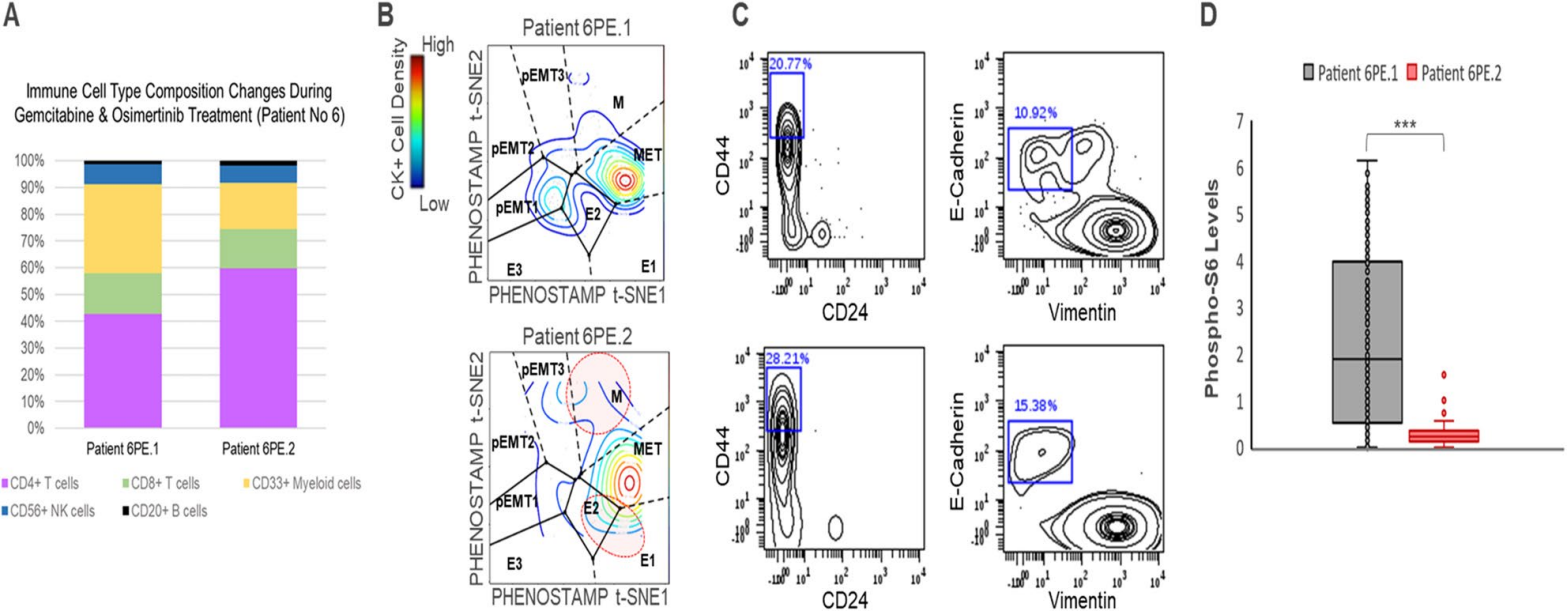
Therapy-induced changes in CK+ cells. (**A**) Immune cell type composition in the PE specimens obtained 2 months apart during therapy from Patient No6. (**B**) Phenotypic changes of CK+ cells from Patient No6 taken 2 months apart (Patient 6PE.1, Patient 6PE.2). Red circles on the bottom density plot denote the increase of cells mapping onto M (mesenchymal and stem-like regions) and epithelial regions (E1, E2) of the map following 2 months of therapy. (**C**) Biaxial CD44/CD24 and E-Cadherin/Vimentin plots of CK+ cells, confirming the increase of CD44hiCD24lo stem-like cells and ECadherin+Vimentin- epithelial cells (%) in the 2nd PE specimen (bottom plots). (**D**) Box and whisker plots depicting phospho-S6 levels (arcsinh-tranformed) in CK+ cells in serial PE specimens from Patient No6. Solid lines indicate median values: 1.88 (n = 284 cells, 6PE.1), 0.23 (n = 39 cells, 6PE.2). ***pvalue < 0.001, Student’s t test). See Supplementary Fig. 7 for additional information.

Because we were able to acquire 2 longitudinal specimens from Patient No6 during treatment, we examined differences between the serial samples (Fig. 5). In terms of the immune cell type composition, there was a decrease in number of CD33+ myeloid cells and increase of CD4 T cells during treatment (32.7 vs 16.6%, 41.9 vs 57.6% respectively, *p* < 0.001, chi-square test) (Figs. 3C, 5A). Number of CK+ cells decreased (4.8% vs 1.51% (Fig. 3A)). In terms of EMT-related phenotypic changes, CK+ cells from the 1st specimen (Fig. 5B, top) mapped predominately onto the MET state; whereas, CK+ cells from the 2nd specimen (Fig. 5B, bottom) mapped with higher densities on the mesenchymal (M) state, and a specific area which we had previously defined as stem-like^11^ (dotted red top circle), and the epithelial states E1 and E2 (dotted red bottom circle). The observed increase of stem-like (CD44hi/CD24lo) and epithelial cells were confirmed in matched CD44/CD24 and E-Cadherin/Vimentin biaxial plots (Fig. 5C). Similar phenotypic state transitions were observed in Erlotinib treated HCC827 cells in in vitro experiments (Supplementary Fig. 7). The most pronounced signaling difference in CK+ cells was observed in phospho-S6 (pS6) expression, which dramatically decreased in specimen 2 (Fig. 5D). pS6 is a signaling component downstream of the EGFR pathway and has been shown to be a readout of Osimertinib activity in both preclinical and clinical studies^28,29^. Therefore, our single-cell analysis suggests a successful therapeutic response in Patient No6 who was undergoing Gemcitabine/Osimertinib treatment.

## Discussion

 Pleural effusions (PEs) are typically collected from advanced stage patients as palliative care, enabling a unique opportunity to monitor their disease status. We present a novel translational approach, whereby using single-cell analysis via mass cytometry and the EMT–MET mapping tool PHENOSTAMP, we study the cellular and molecular profile of PEs from lung cancer patients with a specific focus on characterizing EMT and MET states in CK+ cells and simultaneously assessing immune cell type composition at a personalized level.

Our PHENOSTAMP analysis showed that CK+ cells in PEs are predominantly pEMT or MET-enriched. While EMT and MET have been associated with metastasis and drug resistance, a large proportion of studies that perform phenotypic analysis on clinical specimens, including CTCs and floating malignant cells in PEs, focus only on EMT-related signatures and protein expression^14,15,18,25,26^. In contrast to EMT, MET has been poorly characterized, partly due to the lack of evidence addressing its presence in CTCs and in clinical specimens in general. Prior studies have shown that CTCs that travel in clusters exhibit partial/hybrid EMT features and that this offers greater metastatic potential leading to poor clinical outcome^18,23,25^. We hypothesized that malignant cells derived from PEs of late-stage lung cancer patients, which we identified as CK+ cells, would have a greater chance of exhibiting MET features. To assess EMT and MET-related states in CK+ PE cells, we utilized PHENOSTAMP, a reference EMT–MET map developed previously by our group^11^. One of the advantages of using PHENOSTAMP, is the ability to distinguish malignant cells undergoing EMT from malignant cells undergoing MET. In most studies only a handful of markers (usually low EpCAM, low E-Cadherin and high Vimentin) are used to define the mesenchymal features in, for example, CTCs^24,30^. We however have defined the MET state as a state that has low E-Cadherin and high Vimentin, but one that is distinguished by high MUC1 expression, a glycosylated epithelial marker that has been shown to drive MET in nephrogenesis^31^. Therefore, where several studies support the notion that CTCs exist in a partial EMT or mesenchymal state, we show that a significant number of CK+ epithelial cells in PEs (but not in primary tumors) are in an MET state. Previously, this was shown in a study of small cell lung cancer^32^ where the investigators acquired CTCs from patients and then expanded them in vitro prior to characterization, as opposed to this study where we process and fix PE CK+ cells at time of acquisition.

The causal mechanisms associated with the MET cell state in PEs are poorly understood and necessitate further investigations. We hypothesize that MET in PE CK+ cells may be the result of (1) cells being distant from the primary tumor microenvironment, (2) the metastatic niche, (3) response to treatment, or (4) a combination of these factors. Despite the sample size of our study, we note that only patients who were undergoing treatment at the time of sample collection had the largest fraction of CK+ cells in the MET state (Patients No5 and 6). Studies have shown that various treatments can induce MET in cancer cells^33,34^, and in certain cases this transformation is favorable when it re-sensitizes cells to certain pharmacological agents. However, MET has been regarded as necessary for metastasis, and perhaps this association is consistent with our observation that one of the two patients in our cohort with no MET-enriched CK+ cells was the patient who was initially staged as Stage IIIA (T4N0M0) and had not undergone treatment in the prior 2 months (Patient No7, Fig. 3B). Patient No8 was the other patient with no MET-enriched CK+ PE cells, and this patient was treatment naïve. Instead, patients No7 and 8 had pEMT1-enriched CK+ PE cells. pEMT has been reported in lung cancer CTCs^26^, and it is considered to provide growth and metastatic advantages.

In our study, association between treatment and enrichment of the MET state in PEs appears to be related to the high expression of MUC1. MUC1 has been shown to be expressed in CTCs in ovarian cancer, including EMT-like CTCs that were enriched by platinum-based therapy^35,36^. In metastatic breast cancer, MUC1-positive CTCs were shown to predict chemotherapeutic efficacy; specifically, patients with decreased MUC1 after chemotherapy had a significantly longer progression free survival (PFS)^37^. MUC1 has been demonstrated to drive drug resistance by upregulating multidrug resistance genes in pancreatic cancer^38^, and by enhancing survival pathways in NSCLC cells^39^. MUC1 also confers protection to anoikis in epithelial cells^40^, a critical feature since it protects CTCs from a type of cell-programmed death that initiates upon detachment from extracellular matrix. Therefore, MUC1 may not only help evaluate EMT and MET-related phenotypic changes and response to therapy in PE CK+ cells, but may also serve as a therapeutic target in lung cancer^41^.

In one of the patients with MET-enriched CK+ cells, we analyzed a serial PE specimen drawn 2 months apart while the patient was undergoing chemotherapy and targeted (osimertinib) therapy (Patient No6). In this case, we observed simultaneous enrichment of mesenchymal and epithelial states after 2 months of treatment (Fig. 5B). Interestingly we observed a similar phenomenon in a NSCLC cell line treated with another TKI, Erlotinib (Supplementary Fig. 7). These observations suggest that treatment may induce or select for various EMT states that may be different in their signaling and phenotypic properties, offering more possibilities for cells to obtain survival benefit under various conditions. These types of insights are needed to understand how resistance develops and how one could begin to target and/or predict heterogeneous populations and/or clones of resistant cells. In the specific case, we observed enrichment of a stem-like cell population which has been reported as a drug resistance mechanism in the literature^42^ alongside a population of cells with increased epithelial features. However, our data and analysis are not sufficient to conclude whether these changes were caused by treatment. A larger cohort study of clinical longitudinal data is necessary for delineating the causal relationship between patient therapy and phenotypic state transitions in tumor cells.

Our analysis showed a high correlation between the numbers of CD33+ myeloid and CK+ cells in PEs. Given the immunosuppressive role MDSCs in the tumor microenvironment^27^, we tested whether the CD33+ cells we detected expressed MDSCs markers (CD11b+, HLA-DR-). We were able to identify MDSCs in one of the two patients with an MET enriched CK+ PE (Patient No5). Interestingly, certain myeloid subpopulations have been reported to promote MET at metastatic sites^43,44^, thus, this finding warrants further investigation in a larger cohort and additional markers to better define myeloid subpopulations. Another interesting observation was the presence of proliferative B cells in CK+ PEs versus CK- PEs. Although not much is known on the role of B cells in lung cancer, it has been shown that naïve B cells support MPE formation in lung cancer patients^45,46^. Given the importance of immunotherapy in NSCLC^47^, it is critical in future studies to combine our approach with functional subtyping of various immunosuppressive immune cells (e.g. CD8 activation, Tregs) and their relation to various EMT states in the tumor microenvironment for a more comprehensive immune profiling of primary tumors, liquid biopsies and metastases. This would provide invaluable insights on the progression of primary lung tumors to distant metastases.

Our study presents certain limitations. Although we assume that the majority of CK+ cells are malignant, we cannot exclude the possibility that a proportion of these cells are mesothelial cells that shed in the pleural cavity. Future studies should include additional markers to help distinguish mesothelial from malignant cells (e.g. calretinin^48^). Nevertheless, there are numerous studies that utilize similar approaches to study CTCs in circulation and in PEs. CELLSEARCH, is an FDA approved technology for isolating CTCs from blood that has also been successfully implemented in MPEs^49^. It uses an EpCAM antibody capture system and cytokeratin antibodies for identifying tumor epithelial cells. In most cases CTCs are further enriched by immune, CD45-negative selection^50–52^. Some of these studies confirm CTC identities by subsequent genetic and mutation analyses, and have reported high detection rates for the antibody-capture systems^50^. Furthermore, E-Cadherin as well as MUC1 and MUC4 have been reported to help distinguish malignant from mesothelial cells^53–55^; given the expression patterns of these markers among the EMT spectrum and the fact that we did not detect CK+ cells in four out of nine PE specimens may suggest that only a small % of the CK+ cells we studied here are mesothelial. Filtering MPEs and analyzing single cell suspensions with mass cytometry may miss certain EMT phenotypes associated with intact tumor cell micro-clusters. We are reassured that we are still capturing informative degree of heterogeneity as we can identify a spectrum of EMT states (E, pEMT, M and MET) in CK+ cells detected in liquid biopsies with mass cytometry. Finally, we acknowledge that our feasibility study is limited in terms of sample size and lack of appropriate study controls. Given that pleural effusions are not present in healthy individuals, one way to address this in the future would be to assess cellular phenotypes in PEs in individuals with infections or inflammatory conditions^56^. Scaling our approach in a longitudinal setting on a large cohort with suitable sample replicates and power calculations is warranted for assessing clinical translation of our findings. 

In summary, we present a feasibility study, where we interrogate the cellular profile of PEs in NSCLC patients and provide insights on the EMT-related phenotypic changes of individual cells at a personalized level. By using the EMT–MET reference map PHENOSTAMP, we show that CK+ cells found in PEs are in most cases enriched for pEMT and/or MET states, and that these states may be relevant to disease stage and therapy response. These observations justify investments toward personalized and precision medicine, whereby identifying phenotypic changes at the level of single cells from MPEs, one could better assess disease progression, response to therapy and tailor therapeutic strategy for the individual patient.

## Methods

### Acquisition of clinical specimens

Stanford IRB approved all clinical aspects of this study. Collection and use of patient tissue specimens were in accordance with the Declaration of Helsinki guidelines for the ethical conduct of research and was in compliance with data protection regulations regarding patient confidentiality. Protocols were approved under Stanford IRB protocols #15166 (primary tumors) and #21319 (pleural effusions). All patients provided a written informed consent. For the pleural effusion study patients, inclusion criteria included: (1) Histologically proven diagnosis of NSCLC, small cell lung cancer (SCLC), thymoma, thymic carcinoma, mesothelioma, neuroendocrine tumor, or carcinoma of unknown primary consistent with the presentation of a primary thoracic malignancy. Patients clinically suspected to have a thoracic malignancy were also eligible while undergoing workup and treatment. For this study specifically, only pleural effusion specimens from NSCLC patients were analyzed. (2) Patients were at least 18 years or older and (3) Patients showed ability and willingness to sign a written informed consent document (or had a legally authorized representative to do so on the participant’s behalf). There were no exclusion criteria for this study’s participants.

### Processing pleural effusion specimens for mass cytometry

Fresh pleural effusions (PEs) from 8 late-stage NSCLC patients were obtained from the Stanford Chest Clinic in Palo Alto, CA (Table 1) and placed in 4 °C until processing. In the few cases where PEs specimens were left in 4 °C overnight, no significant effect was observed in viability as assessed with Trypan Blue counting. Specimens were centrifuged at 400×*g* for 10 min to collect cellular material in pellets. Cellular pellets were resuspended in 10% FBS RPMI media and cell suspensions were applied to a MACS 70μM cell strainer for filtration (SmartStrainer, Miltenyi, #130-098-462). Cell strainers were washed once with RPMI and filtered cell suspensions were centrifuged at 400×*g* for 7 min. When PEs were bloody, red blood cells were removed using the Red Blood Cell Lysis Solution ((Miltenyi, #130-094-183). Following red blood cell lysis, samples were washed with RPMI media and centrifuged at 300×*g* for 7 min. Pellets were then resuspended in 1–2 mL of RPMI media, at which point we proceeded to cell count and perform initial % viability assessment using Trypan Blue exclusion. For assessing viability with mass cytometry, cell pellets were subsequently incubated in 1mL PBS containing cisplatin (Sigma-Aldrich #P4394, final concentration 0.5 μM) for 5 min at room temperature. To quench cisplatin reactivity we added 10% FBS RPMI media and centrifuged cell suspensions for 5 min at 500×*g*. Cell pellets were resuspended in RPMI media to reach 0.5–1 × 10^6^ cells/mL aliquots per sample and fixed by adding PFA at a final concentration of 1.6% for 10 min at room temperature. Samples were centrifuged and washed twice at 500×*g* for 5 min at 4 °C to remove PFA with Cell Staining Media (CSM, 0.5% w/v BSA, 0.02% w/v NaN_3_ in PBS). Finally, cell pellets were resuspended in CSM and stored at − 80 °C until multiple PE specimens were collected.

### Primary tumor dissociation and processing for mass cytometry

Following resection from five NSCLC patients, fresh tumor specimens were immersed and transferred from Stanford Hospital to the laboratory in MACS Tissue Storage Solution (Miltenyi, #130095-929) or Phosphate Buffered Saline (PBS) on ice. After recording tumor weight, obtaining macroscopic pictures and removing fat and necrotic areas, tumors were cut into pieces of 2–4 mm. Tumor dissociation was achieved by using the MACS Tumor Tissue Dissociation Kit (Miltenyi, #130-095-929) as per the manufacturer’s instructions and as previously described^11^. Cell count and % viability were assessed as described above.

### Mass cytometry antibodies

Antibodies used for mass cytometry analysis, including information on antibody clone, vendor, metal isotope, and staining concentration, are summarized in Supplementary Table 1. Additional information on when a specific group of antibodies was used in certain runs is described in Supplementary Information. Antibodies were either purchased conjugated from Fluidigm or conjugated to metal isotopes in-house using the MaxPar Antibody Conjugation Kit (Fluidigm) and titrated to determine optimal staining concentrations.

### Mass-tag barcoding and antibody staining for mass cytometry

To improve staining consistency, samples were palladium barcoded and pooled for staining as previously described^11^. Briefly, different combinatorial mixtures of palladium-containing mass-tag barcoding reagents in dimethyl sulfoxide were added to each clinical specimen that was previously resuspended in PBS-saponin solution and mixed with pipetting. Barcoding reagents were added to samples for a 15 min incubation at room temperature. Reaction was quenched with the addition of CSM, followed by several washes with CSM prior to pooling all samples together to proceed with staining. Not all samples described in the study were barcoded, pooled and stained at once. However, all primary tumor samples were barcoded and pooled with PEs from patients No1, 2, 3 and 7. PEs from patients No5 and 8 (Fig. 4B) and 2 PEs acquired from patient No6 (Fig. 5) that were compared in this study were also barcoded, stained and run simultaneously in respective mass cytometry experiments. Antibody staining was performed as previously described^11^. For primary tumors, separate staining cocktails using the same concentrations were prepared with the addition of antibodies towards FAP, and CD31 for gating out stromal, and endothelial tumor populations, respectively. For the mass cytometry run, cells were washed with CSM, twice with filtered double-distilled water, and finally resuspended (~ 10^6^ stained cells/mL) in filtered double-distilled water that contained normalization beads (EQ Beads, Fluidigm). Pooled filtered cell samples were kept on ice throughout the entire run and introduced into the CyTOF 2 (Fluidigm) using the Super Sampler (Victorian Airship and Scientific Apparatus, Alamo, CA, USA). Apart from antibody metal isotopes listed in Supplementary Table 1, event length, barcoding channels (^102^Pd, ^104^Pd, ^105^Pd, ^106^Pd, ^108^Pd, ^110^Pd), normalization beads (^140^Ce, ^151^Eu, ^153^Eu, ^165^Ho, ^175^Lu), DNA (^191^Ir and ^193^Ir), and dead cells (^195^Pt and ^196^Pt) were also recorded.

### Mass cytometry data processing and UMAP analysis

For normalization and single-cell debarcoding respective algorithms were utilized as described previously^11^. Data transformation was achieved by using the inverse hyberbolic sine (ArcSinh) function with a cofactor of 552. Debarcoded samples were uploaded as separate FCS files and analyzed on Cytobank. Dead (cisplatin-positive) and apoptotic (cleaved Caspase-3) cells were removed for all subsequent single-cell analysis (Supplementary Fig. 1). Cytobank software was used for traditional cytometry statistics and visualization (biaxial density plots) and for UMAP dimensionality reduction analysis. All data presented for primary tumors and PEs contained 23,380–127,905 and 13,205–172,669 viable, non-apoptotic single-cell events, respectively, per clinical specimen. We ran 2 separate UMAP analyses. One for all primary tumor samples and one for all PE specimens. 10,000 viable, non-apoptotic cells were sub-sampled per clinical specimen in both runs for clustering and visualization. Markers CD3, CD4, CD8, CD20, CD33, CD56 and Cytokeratins 7 and 8 were used for clustering in both analyses with the addition of FAP and CD31 for the primary tumor clustering analysis (Supplementary Figs. 1–4).

### Projection of cytokeratin positive CK+ cells onto PHENOSTAMP

Construction, algorithm description of PHENOSTAMP and projection of mass cytometry onto PHENOSTAMP using a trained neural network has been described in detail in our previous work^11^. In brief, our trained neural network was used to project manually gated CK+ cells from five primary tumor specimens and five PEs (Supplementary Fig. 1). A *k*-nearest neighbor classification on the partition centers was carried out for each sample to estimate the densities of cells in various EMT state regions on the map. For comparison, we also projected UMAP-defined CK+ cells, and these were in agreement with the projection observations of all manually gated CK+ cells (Supplementary Fig. 8).

### Statistical analysis

Differences between proportions of immune cell types among tumor and PE specimens were assessed using the chi-square test. Signaling differences (mean expression ± S.E) among groups of cells were assessed with the unpaired Student’s *t* test.

### Supplementary Information

Supplementary Information.

## Data Availability

The raw mass cytometry data (.fcs files) from NSCLC patient primary tumors and pleural effusions have been deposited in the Cytobank database under the name “Karacosta et al. mass cytometry data from NSCLC primary tumor and pleural effusion patient specimens” [https://premium.cytobank.org/cytobank/experiments/482121]. All other data that support findings of this study are available within the article and in the Supplement and from the corresponding author upon reasonable request. PHENOSTAMP code availability has been previously published and deposited on GitHub under [https://github.com/anchangben/PHENOSTAMP].
