# Supplementary material for: Phenotyping EMT and MET cellular states in lung cancer patient liquid biopsies at a personalized level using mass cytometry
Source: Sci Rep. 2023 Dec 8;13:21781. doi: 10.1038/s41598-023-46458-5 (PMC10709404; doi:10.1038/s41598-023-46458-5)
Supplement: Supplementary file 1 — Supplementary Information. [file 41598_2023_46458_MOESM1_ESM.docx]

Supplementary Information

**Phenotyping EMT and MET cellular states in lung cancer patient liquid biopsies at a personalized level
using mass cytometry**

Karacosta et al.

**Supplementary Table 1:** Mass cytometry antibody panel used for analyzing primary tumor and pleural effusion NSCLC specimens described in this study. Gray-shaded antibodies were not used in all mass cytometry experiments, but in subset of sample runs. CD31 and FAP antibodies were only used for the primary tumor mass cytometry run (Figure 2). CD45 staining was inconclusive in the primary tumor mass cytometry run and therefore was not used for gating and subsequent analyses (see Supplementary Fig.1 for additional information). Additional antibodies probing for other markers (CD25, non-phosphorylated b-catenin, Notch, phospho-Src, RUNX1, CD69, CD104 and CD15) were used in various runs but gave inconclusive and/or no staining results, therefore are not included in this table or downstream analysis.


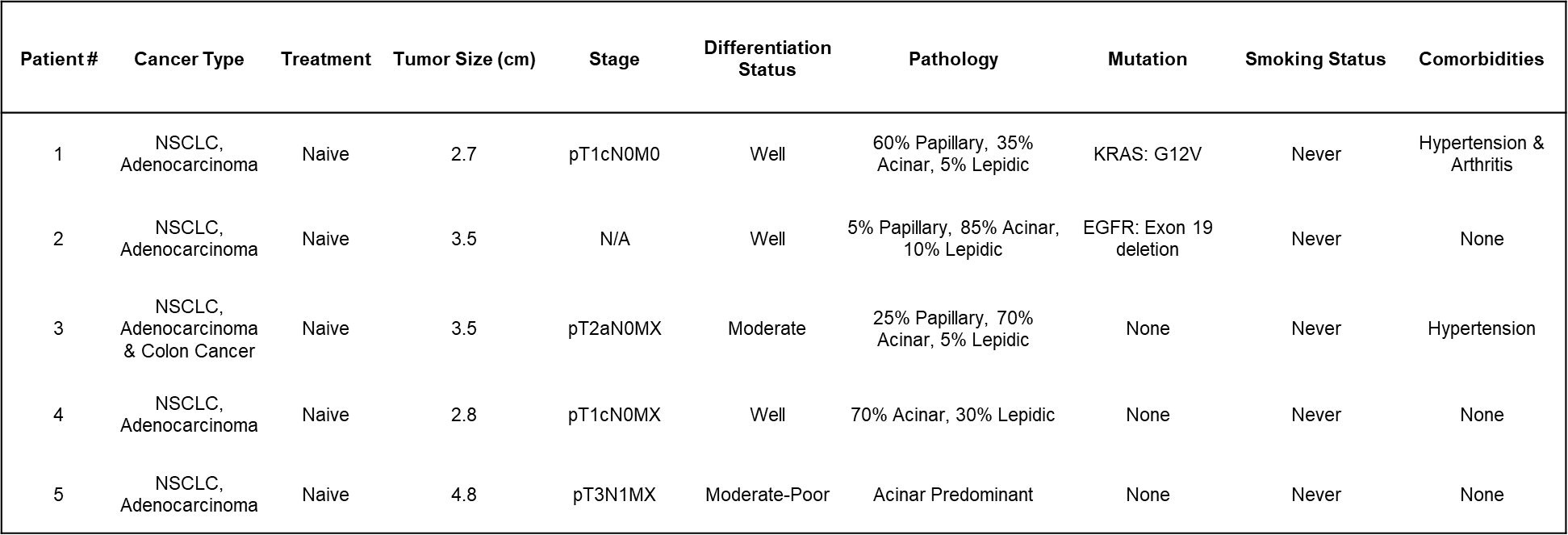


**Supplementary Table 2:** Clinical information and tumor characteristics from the five early-stage, treatment-naïve NSCLC patients described in Figures 1 and 2.


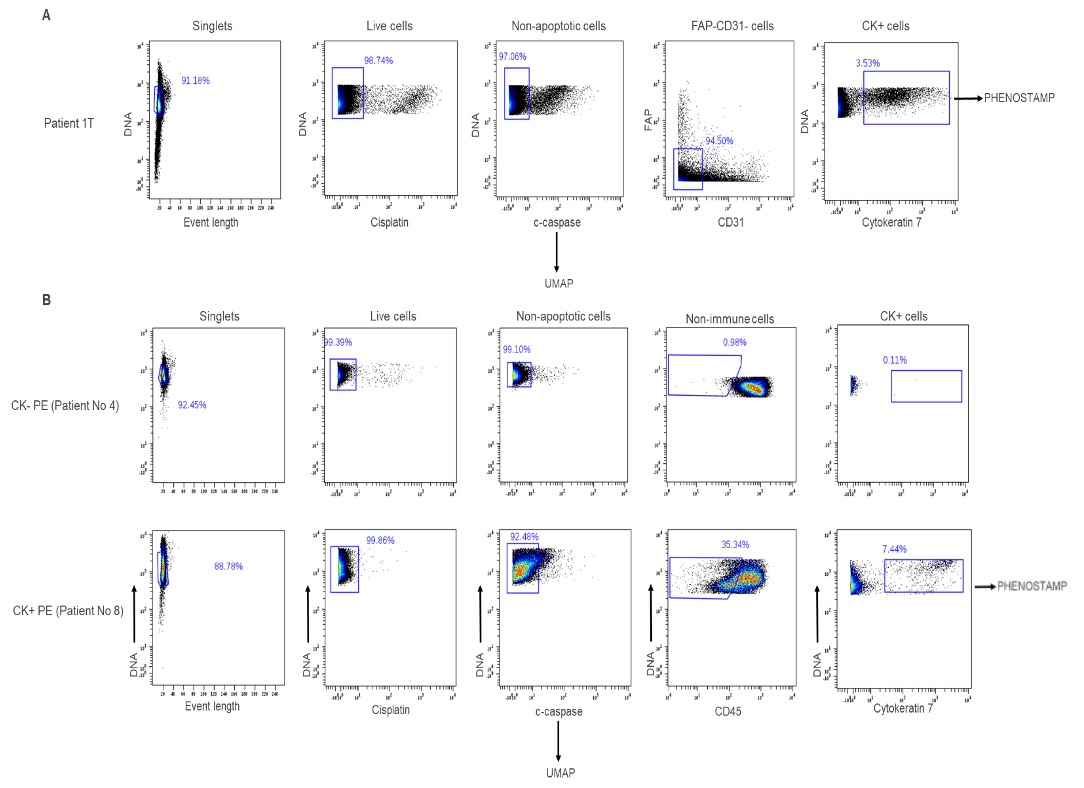


**Supplementary Figure 1:** Gating strategies and downstream analysis of clinical specimens. A) Gating strategy and downstream analysis for primary tumors analyzed with mass cytometry. Representative sample shown, tumor from Patient No 1 (Patient 1T). B) Gating strategy and analysis of pleural effusions (PEs) analyzed with mass cytometry. Shown here are examples of a PE that was negative for Cytokeratin (CK) cells (CK- PE, Patient No4, top) and a PE that was positive for CK cells (CK+ PE, Patient No8, bottom). Briefly, following de-barcoding, manual gating of DNA and cell length parameters were used to separate single cells from debris and doublets. Cisplatin and cleaved-caspase-3 staining was used to remove non-viable and apoptotic cells respectively. UMAP^1^ analysis: UMAP analysis was performed on non-apoptotic cell populations by using the following markers: CD3, CD4, CD8, CD20, CD56, CD33 surface markers (immune cells), Cytokeratins 7 and 8 (CK+ epithelial cells), FAP (stromal cells) and CD31 (endothelial cells). UMAP was performed separately on primary tumors and PE specimens (CD31 and FAP were omitted from the UMAP analysis on PE specimens). Due to CD45 staining discrepancies during the mass cytometry run for primary tumors and one PE specimen (Patient 7PE), CD45 was not used for UMAP analysis and CD45 negative gating could not be implemented. Expression profiles of all clustering markers for each specimen are shown in Supplementary Figures 2-4. PHENOSTAMP^2^ projections: For primary tumors and Patient 7PE we gated CK7+ cells after eliminating CD31+ endothelial and FAP fibroblast cells and projected these onto PHENOSTAMP. For all other PE specimens, we manually gated CD45-CK7+ cells and projected them onto PHENOSTAMP for assessing EMT/MET status. For comparison, we also projected CK
+ cells as defined by our UMAP analysis on PHENOSTAMP and observed no significant differences in EMT/MET status from when we projected manually gated CK+ cells (see Supplementary Figure 8).


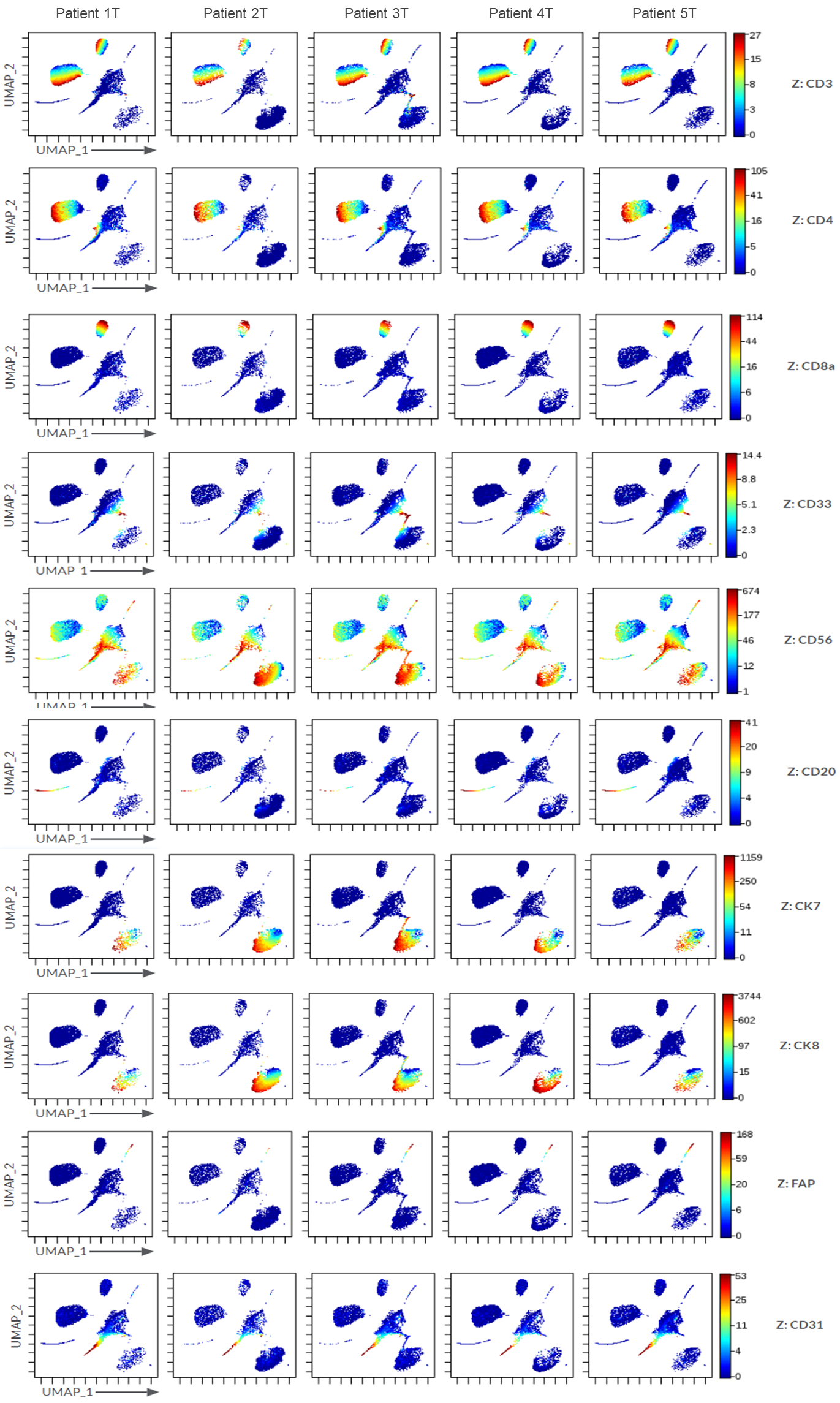


**Supplementary Figure 2:** Expression profiles of all clustering markers in primary tumor specimens (related to Figure 2).

**
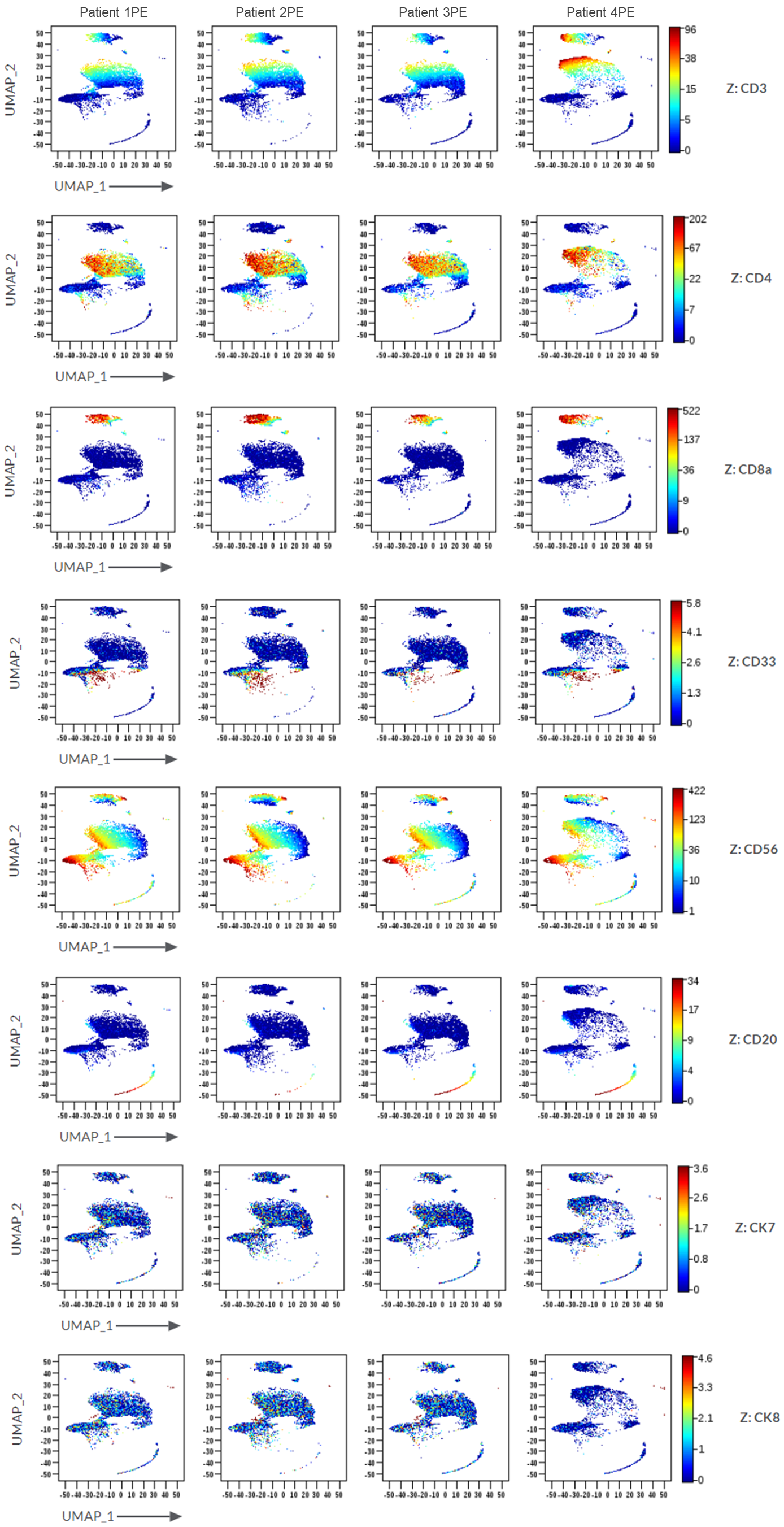
**

**Supplementary Figure 3:** Expression profiles of all clustering markers in PE specimens where CK+ cells were not detected (Patients No 1-4, related to Figure 3).

**
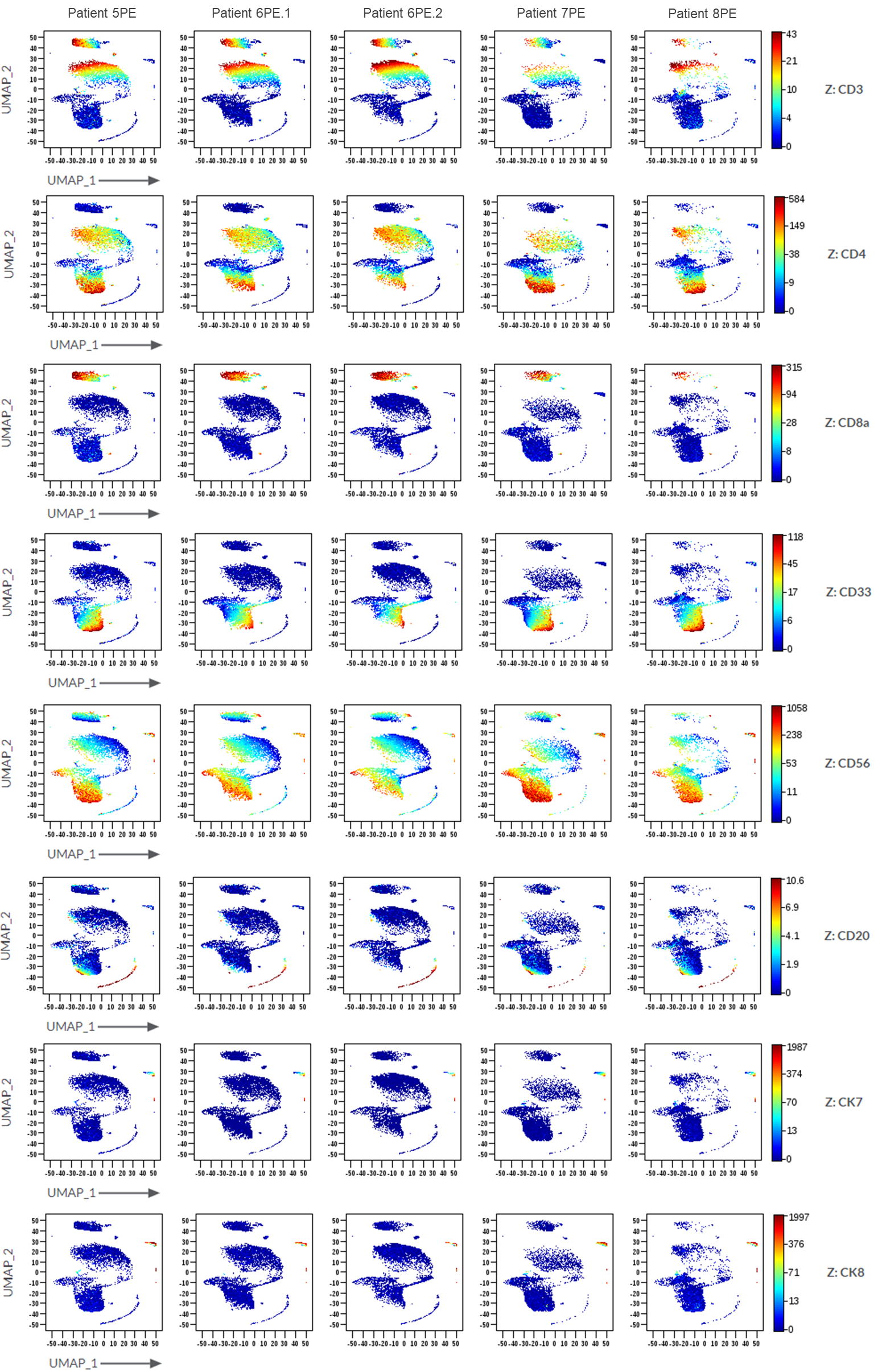
**

**Supplementary Figure 4:** Expression profiles of all clustering markers in CK+ PE specimens (Patients No 5-8, related to Figure 3).

**
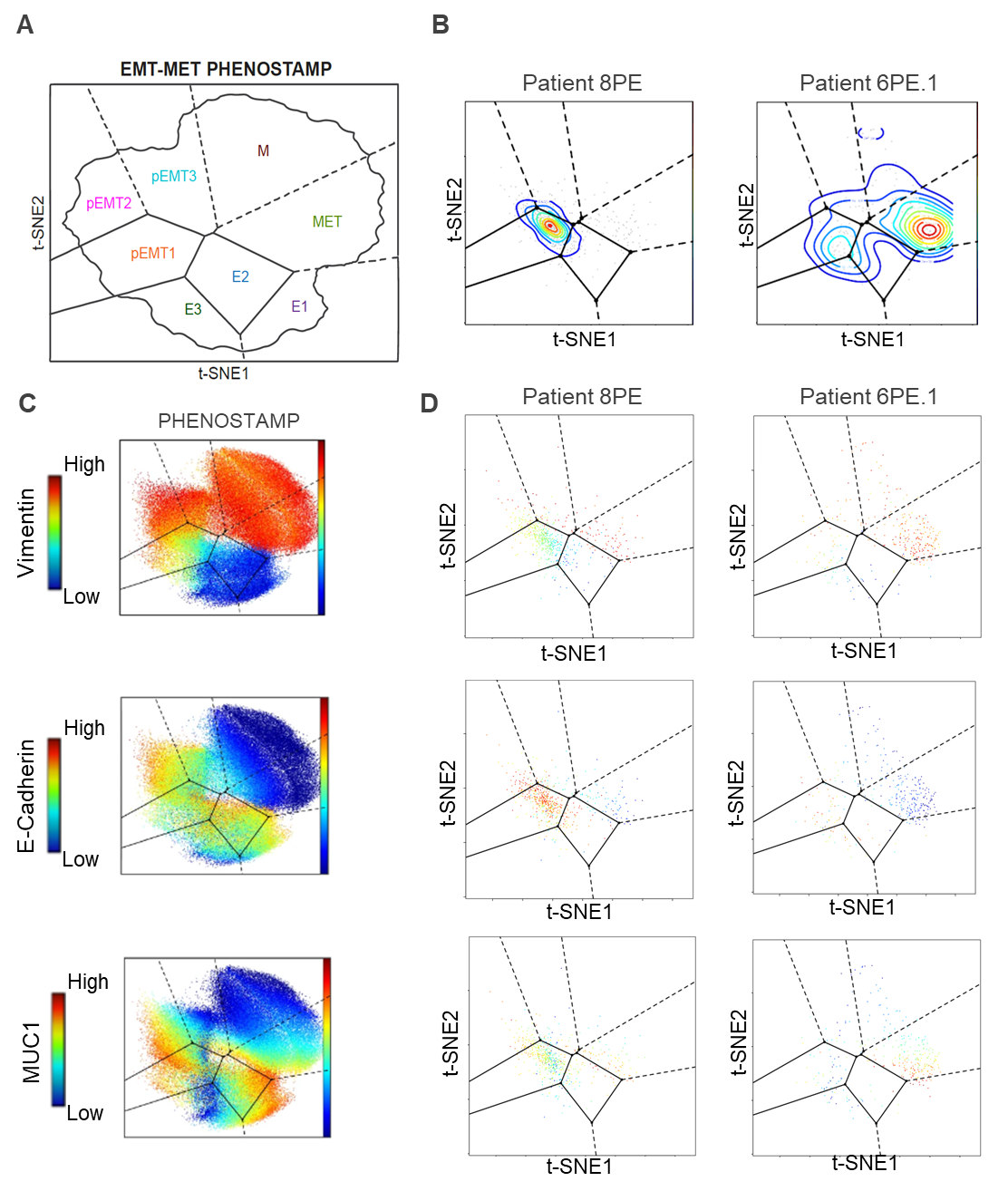
**

**Supplementary Figure 5:** Expression profiles of Vimentin, E-Cadherin and MUC1 in pEMT and MET-enriched PE CK+ cells. A) Schematic diagram of EMT and MET states on PHENOSTAMP^2^. B) Projections of CK+ cells found in Patient 8PE (pEMT-enriched) and Patient 6PE.1 (MET-enriched) on PHENOSTAMP as examples. C) Expression profiles of Vimentin, E-Cadherin and MUC1 in EMT-MET states as observed in the NSCLC lung cancer cell line HCC827^2^. Expression profiles of Vimentin, E-Cadherin and MUC1 in CK+ projected cells from Patient 8PE and Patient 6PE.1.

**
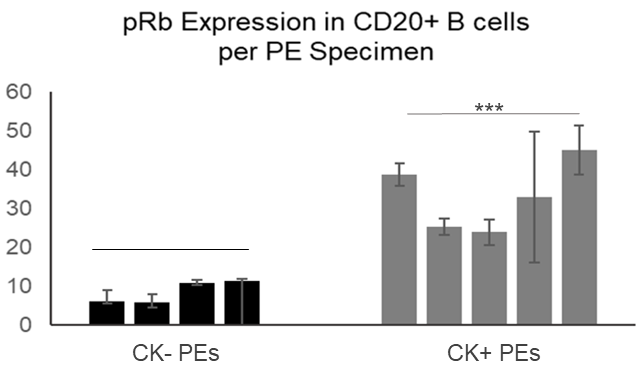
**

**Supplementary Figure 6:** Phospho-Rb (pRb) expression in CD20 B cells is significantly higher in CK+ PEs compared to CK- PEs. Shown are raw mean values (± S.E) of pRb expressed in B cells of each PE specimen. ***p value < 0.001, unpaired Student’s *t* test. B cell numbers from left to right: 261, 23, 335, 815, 236, 111, 158, 30, 59.

**
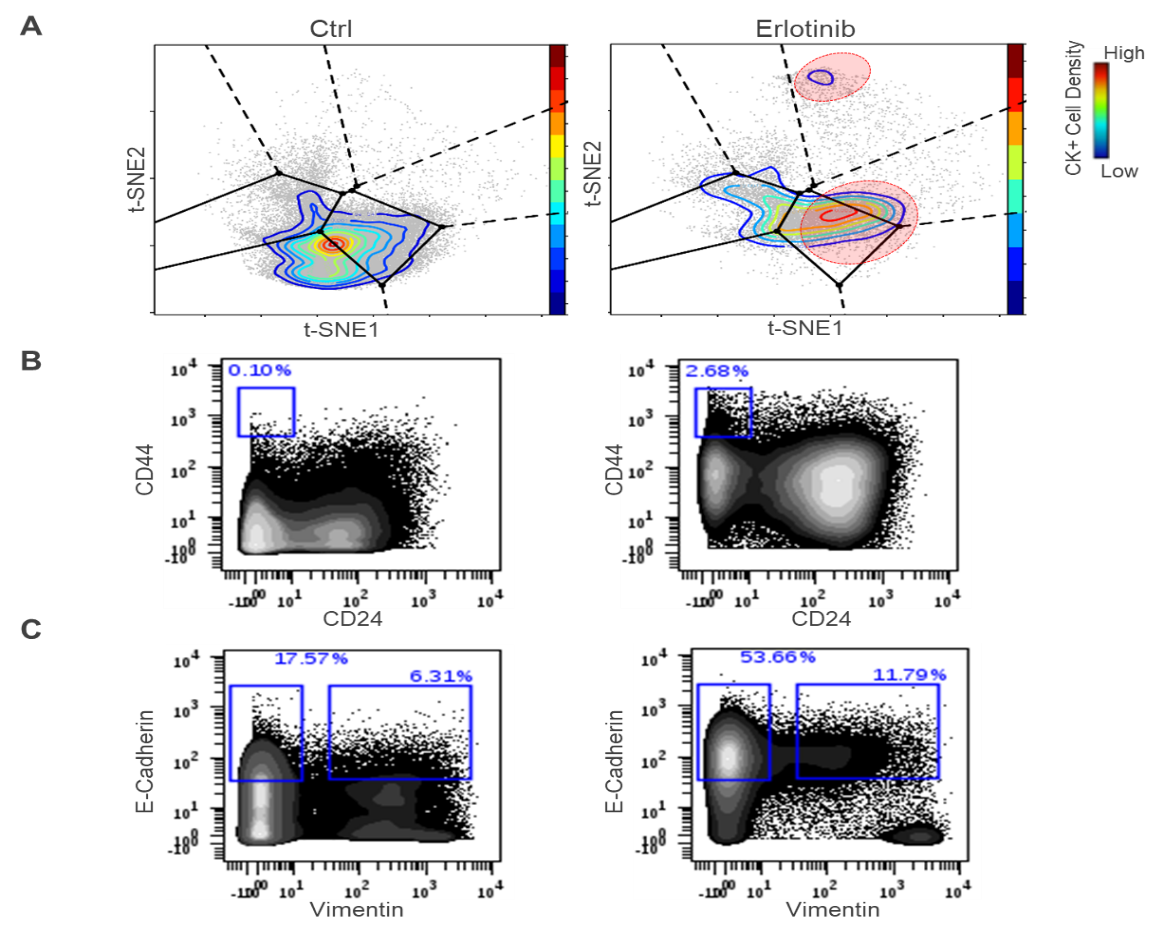
**

**Supplementary Figure 7:** Phenotypic state transitions in Erlotinib treated HCC827 cells. Related to Figure 5. HCC827 cells were treated with 4μM Erlotinib for 15 days. Control and Erlotinib - resistant cells were collected, processed and stained for mass cytometry analysis. A) Projection of cells onto PHENOSTAMP. Note the increase of number of cells in stem-like and Epithelial areas of the map in presence of Erlotinib, similar to what was observed in Patient 6PE.2 during Osimertinib treatment (Figure 5, additional details in main text). B) CD44/CD24 biaxial plots confirm the increase of CD44hi/CD24lo stem-like cells during Erlotinib treatment. C) E-Cadherin/Vimentin biaxial plots confirm the increase of Epithelial (E-Cadherin+ Vimentin- cells during treatment.


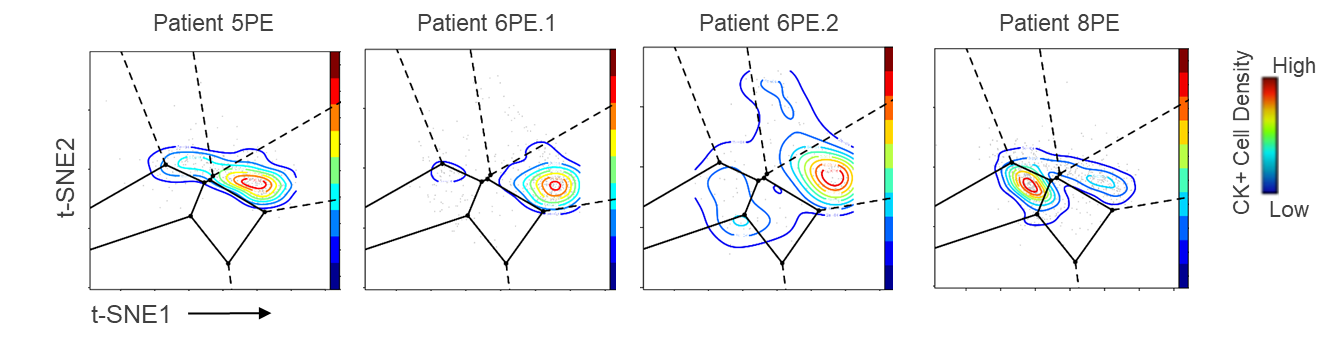


**Supplementary Figure 8:** Projections of UMAP-defined PE CK+ cells are in agreement with projections of manually gated CD45-CK+ cells in terms of pEMT and MET-enriched CK+ cell phenotypes in PE specimens.

**References**

1. Becht, E. *et al.* Dimensionality reduction for visualizing single-cell data using UMAP. *Nature Biotechnology* **37**, 38–44 (2019).

2. Karacosta, L. G. *et al.* Mapping lung cancer epithelial-mesenchymal transition states and trajectories with single-cell resolution. *Nature Communications* **10**, 5587 (2019).
